# Independent Predictors of Late Presentation in Patients with ST-Segment Elevation Myocardial Infarction

**DOI:** 10.5935/abc.20180178

**Published:** 2018-10

**Authors:** Juliane Araujo Rodrigues, Karina Melleu, Márcia Moura Schmidt, Carlos Antonio Mascia Gottschall, Maria Antonieta Pereira de Moraes, Alexandre Schaan de Quadros

**Affiliations:** Instituto de Cardiologia / Fundação Universitária de Cardiologia - IC/FUC, Porto Alegre, RS - Brazil

**Keywords:** ST Elevation Myocardial Infarction, Emergency Medical Services, First Aid, Time Factors

## Abstract

**Background:**

In patients with acute ST-segment elevation myocardial infarction (STEMI), the time elapsed from symptom onset to receiving medical care is one of the main mortality predictors.

**Objective:**

To identify independent predictors of late presentation in patients STEMI representative of daily clinical practice.

**Methods:**

All patients admitted with a diagnosis of STEMI in a reference center between December 2009 and November 2014 were evaluated and prospectively followed during hospitalization and for 30 days after discharge. Late presentation was defined as a time interval > 6 hours from chest pain onset until hospital arrival. Multiple logistic regression analysis was used to identify independent predictors of late presentation. Values of p < 0.05 were considered statistically significant.

**Results:**

A total of 1,297 patients were included, with a mean age of 60.7 ± 11.6 years, of which 71% were males, 85% Caucasians, 72% had a mean income lower than five minimum wages and 66% had systemic arterial hypertension. The median time of clinical presentation was 3.00 [1.40-5.48] hours, and approximately one-quarter of the patients had a late presentation, with their mortality being significantly higher. The independent predictors of late presentation were Black ethnicity, low income and diabetes mellitus, and a history of previous heart disease was a protective factor.

**Conclusion:**

Black ethnicity, low income and diabetes mellitus are independent predictors of late presentation in STEMI. The identification of subgroups of patients prone to late presentation may help to stimulate prevention policies for these high-risk individuals.

## Introduction

In patients with acute ST-segment elevation myocardial infarction (STEMI), the time interval between symptom onset and hospital arrival (delta T) is one of the most consistent predictors of mortality.^1^ Most deaths occur at the start of disease manifestation, and in the 40% to 65% of the cases, death occurs within the first hour, and in 80%, within the first 24 hours.^2^ The benefit of myocardial reperfusion is time-dependent, and the earlier the coronary flow is restored, the better the clinical evolution of the patient.^3^

Although many advances have occurred in the last two decades, resulting in an important impact on morbidity and mortality, the postponing of treatment due to the delay in seeking medical attention is still a major problem in daily clinical practice.^4^ Evidence in the literature indicates that female gender, marital status, Diabetes Mellitus (DM), Systemic Arterial Hypertension (SAH), atrial fibrillation, and age are predictors of hospital arrival delay.^5^^-^^10^

However, there are few contemporary studies evaluating the predictors of late presentation in patients with AMI in the Brazilian setting. The identification of high-risk subgroups of late presentation in the general population could contribute to optimize strategies to reduce the time to access the health care system, with the potential to decrease adverse cardiac outcomes. The aim of this study was to identify predictors of late presentation in patients with STEMI that are representative of daily clinical practice.

## Methods

### Design and population

All patients with STEMI treated at our institution from December 2009 to November 2014 were consecutively and prospectively included. Patients who arrived at the hospital more than 12 hours after symptom onset, those transferred from another health care service and those who refused to participate in the study were excluded.

The study was carried out in accordance with the Guidelines and Norms Regulating Research Involving Human Subjects and was approved by the Institution's Research Ethics Committee.

### Logistics

All patients were interviewed at the time of admission and followed during hospital stay, with clinical, angiographic and laboratory data being collected through a standard questionnaire. The occurrence of cardiovascular events was evaluated by the investigators in up to 30 days after the index event.

### Definitions

STEMI was defined as typical chest pain at rest associated with ST-segment elevation of at least 1 mm of two contiguous leads of the frontal plane or 2 mm in the horizontal plane, or typical pain at rest in patients with a new, or presumably new, left bundle-branch block.^11^

Late presentation was defined as a time interval until hospital arrival of more than 6 hours after the onset of the first STEMI related symptom. Previous heart disease was defined as prior STEMI or previous Percutaneous Coronary Intervention (PCI) or myocardial revascularization surgery (CABG).

Major cardiovascular events (MCVE) were defined as a combination of all-cause mortality, new STEMI or stroke.^11^ New STEMI was defined as recurrent chest pain, elevation of biological markers after the initial natural curve decline, with ST-segment elevation or new Q waves, according to the universal definition of myocardial infarction. Stroke was defined as a new focal neurological deficit with sudden onset, of presumably cerebrovascular cause, irreversible (or resulting in death) within 24 hours and not caused by another readily identifiable cause. The stroke was classified as ischemic or hemorrhagic.^11^

### Patient treatment

The patients were treated according to the institution’s routines, and the researchers did not interfere with any of the applied treatments. All patients with STEMI were referred to coronary angiography and primary PCI (PCIp) as reperfusion therapy, when appropriate, as recommended by the guidelines.^12^ Our institution is a tertiary referral center in cardiology, and the Hemodynamics department operates 24 hours/day, 7 days a week, performing approximately 3,000 coronary angioplasties/year. The emergency department is open to patients who spontaneously seek the hospital, whereas patients who are transferred from other health institutions in the city, the metropolitan region and the countryside of the state are also accepted. In our study, the decisions regarding patient referral from the emergency service to the Hemodynamic laboratory and the percutaneous therapy were left to the attending physicians. Decisions related to the procedure, such as access route, administration of glycoprotein IIb/IIIa inhibitors, aspiration thrombectomy, direct stenting, post-dilatation, models and number of stents used, were made at the discretion of the operators.

The medications used in the initial care followed an institutional routine: aspirin (300 mg), clopidogrel (300 to 600 mg) and anticoagulant (heparin 70 to 100 U/kg) administered at the emergency department immediately after admission.

### Statistical analysis

Data were analyzed using the Statistical Package for Social Sciences (SPSS), version 22.0, and the level of significance of p < 0.05 was considered for all the tests. The Kolmogorov-Smirnov test was used to evaluate data normality. Continuous variables were expressed as mean and standard deviation for those with normal distribution, or as median and 25-75 percentiles. Categorical variables were described as absolute (n) and relative (%) numbers.

The baseline characteristics of patients with late presentation were compared to those who arrived within the first 6 hours using the t-test for independent samples and chi-square test, as appropriate. Univariate and multivariate analyses were performed using the multiple logistic regression method, with late presentation as the dependent variable, and the variables with a p value ≤ 0.20 in the univariate analysis being included in the multivariate analysis.

The WINPEPI program, version 11.43, was used to calculate the sample size, which was calculated as 1,076 patients considering a statistical power of 90%, significance level of 5%, proportion of late presentation of 40% and odds ratio of 1.5 for the female gender as a risk factor.^13^ An addition of 10% was made to control possible losses and refusals, and the final sample size consisted of 1,200 patients.

## Results

Between December 2009 and November 2014, 1,297 individuals met the eligibility criteria and were included in the study. For 302 patients (23%), the time of arrival at the hospital since the chest pain onset was > 6 hours, being considered as late presentation according to the criteria defined in the study protocol.

Table 1 shows the baseline characteristics of the population, according to the presence or not of late presentation. The median time of presentation was 3.0 [1.4-5.5] hours, being significantly higher in those considered as late presentation (8.5 [7.0-11.9] hours vs. 2.2 [1.0-3.7] hours). There was no statistically significant difference in relation to the mean age in the two groups. On the other hand, patients with late presentation were more often women of Black ethnicity with low income and lower educational level, when compared to those who arrived within the time window of the first 6 hours from pain onset.

**Table 1:** Basal characteristics of patients

Characteristic	Total n = 1.297	< 6 horas n = 995	≥ 6 horas n = 302	Valor de p
**Sociodemographic data**				
Female gender	29	26	37	0.001
Age	60 .7 ± 11.6	60 ± 11.7	62 ± 11.5	0.82
Black ethnicity	15	13	19	0.009
Income < 5 minimum wages	72	69	82	< 0.001
Schooling ≤ 8 (years)	52	50	60	0.008
Delta T (hours)	3. 00 [1.40-5.48]	2.16 [1.00-3.70]	8.50 [7.00-11.87]	
**Risk factors for CAD**				
Arterial hypertension	66	65	68	0.37
Active smoking	54	54	56	0.95
Dyslipidemia	37	37	35	0.66
Family history	33	34	33	1.00
Diabetes mellitus	25	23	32	0.001
**Previous medical history**				
Previous CAD*	29	31	23	0.004
Depression	19	18	22	0.19
Stroke	6.1	5.9	6.6	0.75
Heart failure	5.5	3.2	3.6	0.86
Chronic kidney disease	3.3	6.3	2.7	0.02
Killip III/IV	7	6.9	7.6	0.75

Statistical tests: t-test, Mann-Whitney and chi-square test. Results expressed in %, mean ± standard deviation, and median and 25-75 percentiles.

* Previous CAD, acute myocardial infarction or prior myocardial revascularization. CAD: coronary artery disease.

The two groups were overall similar regarding the presence of risk factors for coronary artery disease (CAD), but the percentage of patients with DM was significantly higher among those with late presentation. Regarding the comparisons between pre-hospitalization diagnoses, we observed that patients with late presentation less often had a prior diagnosis of CAD (STEMI or myocardial revascularization) and chronic renal failure, and the frequency of other comorbidities was not statistically different. Regarding the atherosclerotic disease burden, we did not observe statistically significant differences between the groups related to the time of clinical presentation. Most patients with late presentation had lesions in one vessel (48%), 31% had lesions in two vessels and 19% in three vessels - similar rates to those without late presentation (49%, 31% and 18%, respectively; p = 0.72).

Table 2 shows the odds ratios of the clinical characteristics and late presentation, before and after adjustment by multiple logistic regression analysis. The independent predictors of late presentation were Black ethnicity, income less than five minimum wages and DM, whereas prior CAD was a protective factor.

**Table 2:** Uni- and multivariate analysis of characteristics associated with late presentation

Variables	OR (95%CI)	p value	Adjusted OR (95%CI)	p value
Female gender	1,42 (1,16-1,74)	< 0,001	1,13 (0,90-1,42)	0,28
Age	1,00 (0,99-1,01)	0,99	1,00 (0,99-1,01)	0,99
Black ethnicity	1,41 (1,10-1,79)	0,005	1,43 (1,11-1,84)	0,005
Income < 5 minimum wages	1,81 (1,37-2,40)	< 0,001	1,60 (1,19-2,15)	0,001
Schooling ≤ 8 years	1,33 (1,08-1,65)	0,007	1,05 (0,84-1,31)	0,66
Depression	1,17 (0,92-1,48)	0,19	1,15 (0,90-1,47)	0,25
Diabetes mellitus	1,42 (1,15-1,74)	0,001	1,37 (1,10-1,71)	0,005
Previous CAD *	0,70 (0,55-0,89)	0,004	0,72 (0,55-0,94)	0,02
Heart failure	0,47 (0,24-0,91)	0,02	0,54 (0,26-1,13)	0,10

* Previous CAD, acute myocardial infarction or prior myocardial revascularization. OR: odds ratio; 95% CI: 95% confidence interval; CAD: coronary artery disease.

**Figure 1:**
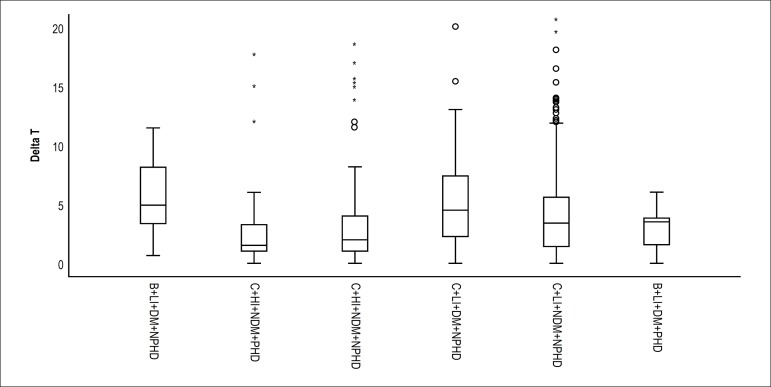
Median time of presentation, according to different combinations of late presentation predictors. C: caucasian; B: black; LI: low income (< 5 minimum wages); HI:high income (= 5 minimum wages); DM: diabetes mellitus; NDM: does not have diabetes mellitus; PHD: previous heart disease; NPC: does not have previous heart disease.

Figure 1 shows the median time of presentation in the patients’ subgroups, according to different combinations of predictors of late presentation, showing a large difference in time regarding a certain combination of predictors. For instance, patients with all predictors of late presentation (Black ethnicity, low-income, DM patients, and no previous cardiovascular disease) had the highest median time of presentation, while those with none of the predictors (Caucasian ethnicity, high income, no DM and previous cardiovascular disease) had the lowest median time of presentation (p < 0.001), as shown in figure 1.

**Figure 2:**
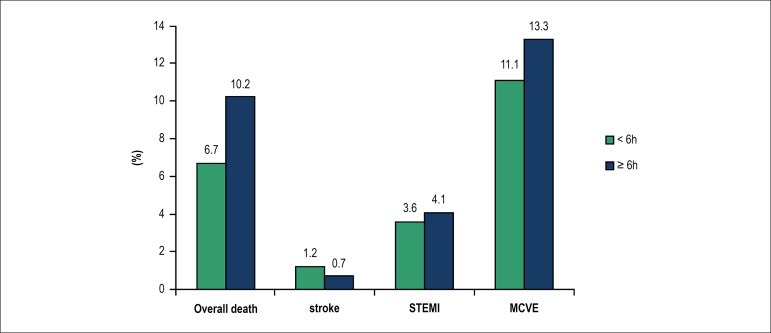
outcomes in 30 days. STEMI: acute ST-segment elevation myocardial infarction; MCVE: major cardiovascular events.

Figure 2 shows the rates of cardiovascular events in 30 days in patients with late presentation or without. Patients with late presentation had significantly higher mortality rates (p < 0.05), and comparisons between groups, considering the occurrence of other clinical outcomes, did not show statistically significant differences.

## Discussion

This study showed that the main predictors of hospital arrival delay in patients with STEMI, treated at a referral hospital in Cardiology in the Southern Region of Brazil, were Black ethnicity, low income and DM, whereas the presence of prior heart disease was associated with earlier arrival. Individuals with all predictors of late presentation had mean time of hospital arrival more than two-fold higher than those who had none of these characteristics. These findings are important, since the time from symptom onset to hospital arrival is one of the main determinants of mortality in STEMI,^3^ as also demonstrated in our study.

Black ethnicity was one of the independent predictors of late presentation in patients with STEMI in the present study. This finding is compatible with data from the CRUSADE (Can Rapid Risk Stratification of Unstable Angina Patients Suppress Adverse Outcomes with Early Implementation) registry, which showed that Caucasians arrive earlier than Blacks while studying a population of more than 100,000 patients (Odds Ratio - OR -2.2; 95% Confidence Interval: 95%CI -4.2 - -0.3; p = 0.03).^14^ On the other hand, a large study involving more than 43,000 consecutive patients with STEMI from the ACTION-GWTG database showed that there is no significant difference regarding time of arrival between Black and Caucasian patients.^15^ Racial differences could be explained by genetic or socioenvironmental characteristics, and we are unaware of studies that have showed differences in pain threshold according to ethnicity. On the other hand, individuals of Black ethnicity in Brazil show unfavorable socioeconomic and cultural status when compared to those of White ethnicity, which could explain our findings.

From this perspective, low-wage income was also identified as an independent predictor of late presentation in our study. Nguyen et al.^8^ performed a systematic review that also showed that patients with low socioeconomic status seek medical attention later.^8^ Low income may be associated with the patient's recognition of their symptoms and pathology, inferring that people with better level of schooling seek emergency services earlier.^8^ On the other hand, Qian et al.^1^ analyzed 100 patients with STEMI in China, with no association of low wage income with late presentation.^1^

In our study, the diagnosis of DM was also an independent predictor of late presentation, which is compatible with the evidence available in the literature.^16^^-^^21^ Patients with DM more frequently have silent ischemia, which may be explained by the presence of diabetic neuropathy and a higher pain threshold.

Previous heart disease was considered a protective factor for late presentation, and the association between this characteristic and the time of presentation varied according to the studies. Kuno et al.^22^ demonstrated that patients who had been previously submitted to a percutaneous coronary intervention procedure had a shorter time of presentation.^22^ In a cross-sectional study that included 335 patients and considered late presentation arriving at the hospital within 12 hours of pain onset, previous STEMI and revascularization did not show a statistically significant association with time of presentation.^23^ Our study did not include analyses of the associated mechanisms between the presence of predictors and the occurrence of late presentation, but it could be speculated that patients who had previous cardiac events or were submitted to myocardial revascularization procedures would be more familiarized and conscious about the disease and the need to seek medical attention quickly.

Women showed significantly longer time until hospital arrival than men, but female gender did not remain an independent predictor of late presentation in the multivariate analysis. The association between female gender and hospital arrival delay after chest pain onset has also been reported in other studies, and it has been found that women more often have atypical symptoms than men.^24^^-^^26^

### Limitations

In this study, we did not have available information regarding the distance between the patients and the hospital when they had the chest pain onset, a fact that may have an influence on hospital arrival delay. However, most of the patients who come spontaneously to our institution are city residents. Because it is located downtown, travel time does not exceed 30 minutes in most cases. It is important to emphasize that patients transferred from other hospitals and health institutions were excluded from our study, since the objective was to analyze the factors that influence spontaneous delay in search for medical care of patients with infarction, and not to analyze factors that have an impact on medical transfer time.

We considered analyzing the association between the distance from the patients' home to our institution, but many patients were not at home at the time of pain onset, but at work or another location, and therefore this analysis was not included in the present report.

We did not have available ventricular function information from all patients, because left ventriculography is not routinely performed during catheterization and primary percutaneous coronary intervention (pPCI) to minimize contrast volume. However, the percentage of patients with previous CHF who presented with Killip III/IV class at the time of STEMI was similar, suggesting that left ventricular function in both groups was not significantly different. This was a single-center study in a large tertiary cardiology hospital, and the results shown herein may not be valid for populations that are significantly different from ours.

## Conclusions

The independent predictors for late presentation to the hospital in patients with acute ST-segment elevation myocardial infarction were Black ethnicity, low-income and DM, whereas a history of previous heart disease was a protective factor. Approximately one-fourth of the patients in this sample were late arriving at the hospital, and their mortality rate was significantly higher than those who arrived early. Patients who had all of the characteristics associated with late presentation showed a two-fold delay related to hospital arrival when compared to those without these characteristics, which illustrates the potential opportunity to decrease the mean time of arrival if public health interventions focused on these high-risk subgroups are carried out.
